# Contamination of a Dental Unit Water Line System by Legionella Pneumophila in the Mashhad School of Dentistry in 2009

**Published:** 2012-06-30

**Authors:** B Ajami, K Ghazvini, T Movahhed, N Ariaee, MT Shakeri, S Makarem

**Affiliations:** 1 Department of Pediatric Dentistry, Dental School, Mashhad University of Medical Sciences, Mashhad, Iran; 2 Department of Mycobacteriology, Ghaem Hospital, Mashhad University of Medical Sciences, Mashhad, Iran; 3 Bu-Ali Research Center, Bu-Ali Research Institute, Mashhad University of Medical Sciences, Mashhad, Iran; 4 Department Community Medicine and Health, School of Medicine, Mashhad University of Medical Sciences, Mashhad, Iran; 5 Student of Dental School, Mashhad University of Medical Sciences, Mashhad, Iran

**Keywords:** Legionella, Dental unit water line system, Contamination, Iran

## Abstract

**Background:**

Dental unit waterline system is considered potential source for contamination with Legionella species. The aim of this study was to determine if contamination of a dental unit water line system by Legionella pneumophila serogroup1 in the Mashhad School of Dentistry occurred in 2009.

**Methods:**

A total of 52 dental units were selected from all clinical departments of the Mashhad School of Dentistry. Samples of water were collected from outlets of water/air spray, high-speed dental hand pieces and water cup fillers. Samples were tested via the ELISA method.

**Results:**

At the beginning of the work day, a total of 36.1 percent of dental units were contaminated by Legionella pneumophila serogroup 1.

**Conclusion:**

Infection control of the dental unit water line system regarding legionella in the Mashhad School of Dentistry is a challenge and engineering controls should be used in contaminated clinics.

## Introduction

Dental unit waterline is considered sources of water-borne opportunistic pathogens, such as Legionella, because dental units contain lengths of narrow bore tubing that offers an optimal environment for devel-oping sessile microbiologic communities.[1]

When a dentist uses a high-speed hand piece, ultrasonic devices or air/water spray, Legionella aerosols are dispersed into the air and can be inhaled, causing serious nosocomial infections, especially in susceptible hosts.[2]

In the Ma'ayeh Study, 86.7% of dental units were infected with Legionella pneumophila (L. pneumophila) at the start of work days;[3] a study in Ahwaz (a city in Iran) showed that Legionella existed in therapeutic equipment, and the greatest contaminated sources were dental units.[4]

L. pneumophila is responsible for over 80% of legionellosis, and of the 16 serogroups of L. pneumophila, serogroup 1 most frequently causes legion-naires disease.[5] Therefore, water quality monitoring in dental settings is essential for the early detection of Legionella and for the prevention of nosocomial legionellosis. This study was designed to investigate contamination of a dental unit water line system by L. pneumophila serogroup 1 in the Mashhad School of Dentistry in 2009.

## Materials and Methods

A total of 52 dental units (three different models) were selected from all clinical departments of the Mashhad School of Dentistry, including oral health and community dentistry, pediatric dentistry, orthodon-tics, oromaxillofacial surgery, periodontics, operative dentistry, endodontics, prosthodontics, the isolated room, operating and the implant room. It was not possible to identify age of dental units. The dental units were selected by a randomized stratified sampling method; we needed one set of 30% of intact dental units in each clinical department that ranged from 1 to all number of intact dental units in each clinical department, for this purpose we used web-based service (randomizer.org). Samples of water were collected from outlets of water/air spray, lubricated and sterilized high-speed dental hand pieces and water cup fillers after the units were out of service for 12-16 hours.

Five water samples (250 ml each) were collected from each unit in a sterile flask. Three water samples were taken from the high-speed dental hand pieces at the following points in time: First, before beginning the work day (8-8.5 am), then after 120 seconds of flushing, and finally, at mid-day (12-12.5 pm) after 20 seconds of flushing.

One water sample was taken from the water/air spray, and one sample was taken from the water cup filler of dental units (8-8.5 am) before beginning the work day. Also, samples were taken from the water input to the dental school and the collector every two weeks, over a period of two months. Before the sample was taken, each of the sampling parts was disinfected with intermediate disinfectant (Deconex solarcept), following the manufacturer’s instructions.

250 ml of all water samples was filtered over 0.2 μm-pore membrane filters (Albet). Filters were put inside the filter holder (Millipore), and passage through the filters was facilitated by a vacuum pump (Rocker 300). Each filter was placed into a test tube containing 1 ml of distilled water, and after shaking the sample was tested by the ELISA method. Following the manufacturer’s instructions, the test was performed with 100 μl samples.

Detection limit for L. pneumophila serogroup 1 in water samples with Bartels’ ELISA test was approx-imately 780 CFU/ml.[6] In our study, water samples were concentrated 250 times; to calculate the number of L. pneumophila serogroup 1 in water samples, 780 CFU/ml was divided into 250 and detection limit of 3.12 CFU/ml for samples were obtained. Therefore, positive samples had at least 3.12 CFU/ml and negative samples had less than 3.12 CFU/ml. According to the Bartels Company instructions, the results were read visually within 15 minutes of running the test by an expert technician who had no connection with the study. The density of colors was judged by using a visual interpretation card that labeled five levels of color intensity. Data analysis were performed with SPSS software (Version 15, USA) by Chi Square test.

## Results

Water samples obtained from the water input and water collector, located on the ground floor and half floor respectively, were negative for L. pneumophila serogroup 1. At the beginning of the work day, a total of 36.1% (19/52) of units in the study were contaminated by L. pneumophila serogroup 1; before 2 minute turbine flushing, 17.3% of turbine outlet samples were infected (9/52); after 2 minute turbine flushing, 5.7% of turbine outlet samples were infected (3/ 52); and at 12 o'clock, only 5.7% (3 / 52) of dental units were infected with L. pneumophila serogroup 1.

The pediatric dentistry, restorative dentistry, endodontics, periodontology, prosthodontics and orthodontics clinics were contaminated at the beginning of daily work. Comparison of turbine contamination at three different times showed that turbine contamination before flushing was significantly higher than contamination after flushing at the beginning of the work day (p=0.02), and turbine contamination at midday (12 o'clock) was significantly lower than contamination at the beginning of the work day (p=0.02).

## Discussion

A total of 36.1% of the dental units at the beginning of the work day were contaminated. Challacombe et al.,[7] concluded that Legionella was present in 25% of 194 units. Bartels ELISA test is a rapid-screening method for the detection of Legionella, but its sensitivity is low,[6] so contamination rate may be higher than what we reported. According to our findings, in positive samples, Legionella was present at concentrations of 312 CFU/100 ml or greater. Exner et al.,[8] Suggested guidelines for the acceptable number of Legionella in water samples. A concentration of 10–1000 CFU per 100 ml of water indicates that the water supply should not be used in medical-technical appliances and that monitoring should be performed twice a year. According to this guide, in contaminated clinics, the use of water equipment should be halted, further studies should be performed and engineering controls should be employed for infection control.

In our study, water samples taken from the water entrance to the dental school and the water collector were free of Legionella. Before and during study period, free residual chlorine in water entrance was measured approximately at 0.5 mg/L and pH of the water was measured at 7.5, using the DPD colorimetric method. In water samples collected from dental units, there was very low free residual chlorine (0 mg/L). Lack of free residual chlorine in water samples obtained from the dental units may be responsible for the presence of a microbial load, including Legionella.

Ma'ayeh et al.,[3] suggested that flushing of DUWL can be a first solution in reducing L. pneumophila counts. We observed a significant decrease of L. pneumophila after flushing but we considered flushing as a temporary solution because biofilm may be separated at any time and dispersed in the air as aerosols, so engineering control is essential and specific disinfecting program for DUWL should be established.
